# 121例手术切除的N2-Ⅲa期非小细胞肺癌患者的生存分析

**DOI:** 10.3779/j.issn.1009-3419.2015.08.06

**Published:** 2015-08-20

**Authors:** 合利 杨, 亮 戴, 培 李, 潞艳 申, 万璞 闫, 梦颖 范, 克能 陈

**Affiliations:** 1 100142 北京，北京大学肿瘤医院暨北京市肿瘤防治研究所胸外一科，恶性肿瘤发病机制及转化研究教育部重点实验室 Department of Thoracic Surgery I, Key Laboratory of Carcinogenesis and Translational Research (Ministry of Education), Peking University School of Oncology, Beijing Cancer Hospital, Beijing 100142, China; 2 010059 贵州，贵州省肿瘤医院乳腺外科 Department of Breast Surgery, Guizhou Province Cancer Hospital, Guiyang 010059, China

**Keywords:** 肺肿瘤, N2淋巴结转移, 生存分析, Lung neoplasms, N2 lymph node metastasis, Survival analysis

## Abstract

**背景与目的:**

N2-Ⅲa期非小细胞肺癌（non-small cell lung cancer, NSCLC）手术抑或非手术仍存颇多争议。我们通过回顾性分析121例手术的N2-Ⅲa期NSCLC患者的生存，探讨N2-Ⅲa期NSCLC患者术后远期生存的因素。

**方法:**

选取北京大学肿瘤医院单医生组肺癌前瞻性数据库2000年1月-2013年6月共1, 290例NSCLC手术患者，其中N2-Ⅲa期NSCLC患者121例。分析性别、年龄、吸烟、围手术期化疗、切口、病理、脉管癌栓、pT分期、肿瘤大小对N2-Ⅲa期患者生存的影响；比较单站N2与多站N2的生存差异；比较术中或术后病理N2者（Ⅲa1/a2）与治疗前N2者（Ⅲa3/a4）的生存差异。单因素分析采用*Kaplan-Meier*法计算生存，*Log-rank*检验。多因素分析采用*Cox*回归分析。

**结果:**

全组121例患者5年生存率为43.6%，中位生存时间50.3个月。单因素分析显示单站N2转移与多站N2转移的5年生存率分别为58.3%和25.5%（*P*=0.001）；Ⅲa1/a2期者与Ⅲa3/a4期者5年生存率分别为52.7%和38.4%（*P*=0.020）。多因素分析显示仅单站N2转移（HR=0.326, 95%CI: 0.186-0.572, *P* < 0.001）与Ⅲa1/a2（HR=0.494, 95%CI: 0.259-0.941, *P*=0.032）是影响本组N2-Ⅲa期患者远期生存的独立因素。

**结论:**

N2-Ⅲa期NSCLC中单站N2转移者预后好于多站N2转移者。Ⅲa1/a2期患者预后好于Ⅲa3/a4期患者。高选择性N2-Ⅲa期NSCLC患者采取以外科手术为主的多学科综合治疗可获得较满意的远期生存。

## Abstract (en)

**Background and objective:**

It has still been controversial to treat N2-Ⅲa non-small cell lung cancer (NSCLC) patients by surgery or non-surgery. We retrospectively analysed the survival of 121 stage N2-Ⅲa NSCLC patients and explored their postoperatively long-term prognostic factors.

**Methods:**

All of 1, 290 patients in Beijing Cancer Hospital underwent resection by single-surgeon-team, among which 121 cases with stage N2-Ⅲa were enrolled in the study. We retrospectively analysed the impact of gender, age, smoking, perioperative chemotherapy, incision, histological type, vascular tumor emboli, pTstage and tumor size on survival of stage N2-Ⅲa patients, and compared the survival between patients with single-and multi-station N2 metastasis, and between intraoperatively or postoperatively pathological N2 (Ⅲa1/a2) and preoperative N2 (Ⅲa3/a4). Univariate analysis was conducted by *Kaplan-Meier* curve, and significance test was performed by *Log-rank* test and Cox regression factor analysis was applicated for multivariate analysis.

**Results:**

The 5-yr of all the 121 cases was 43.6%, with a median survival time being 50.3 mo. Univariate analysis showed the 5-yr of patients with single-and multi-station N2 metastasis were 58.3% and 25.5%, respectively (*P*=0.001), 5-yr of patients with stage Ⅲa1/a2 and stag Ⅲa3/a4 were 52.7% and 38.4%, respectively (*P*=0.020). Multivariate analysis demonstrated that only single station N2 (HR=0.326, 95%CI: 0.186-0.572, *P* < 0.001) and Ⅲa1/a2 (HR=0.494, 95%CI: 0.259-0.941, *P*=0.032) were independent prognostic factors for stage N2-Ⅲa lung cancer patients.

**Conclusion:**

The prognosis of stage N2-Ⅲa NSCLC patients with single-station N2 metastasis were better than those with multi-station N2 metastasis. Besides, Ⅲa1/a2 patients had a better survival compared with stage Ⅲa3/a4 patients. A multi-disciplinary comprehensive treatment based on surgery may allow patients with high selective stage N2-Ⅲa NSCLC to obtain a comparatively satisfying long-term survival.

非小细胞肺癌（non-small cell lung cancer, NSCLC）约占肺癌总数的80%-85%，其中早期NSCLC以手术为主，晚期NSCLC以化疗及靶向治疗为主，而局部进展期的N2-Ⅲa期NSCLC的治疗则颇多争议。N2-Ⅲa期NSCLC是一组异质性非常明显的疾病，既有术后镜下才发现的隐匿性N2，也有影像学上既已肿大的单站N2或多站N2，还包含了多站淋巴结融合成团的N2。Detterbeck等^[1]^认为可把N2-Ⅲa期患者分成预后不同的亚组，N2状态不同预后迥异。根据2003年美国胸科医师学院的分类标准^[2]^，将N2-Ⅲa期分为4类，Ⅲa1期指最后的病理检查偶然发现的N2；Ⅲa2期指术中发现的单站N2；Ⅲa3期指术前分期（纵隔镜、其他的淋巴结活检或正电子发射计算机断层显像（positron emission tomography-computed tomography, PET/CT）发现的单站或多站N2；Ⅲa4期指巨块或固定的多站N2（CT上短径 > 2 cm）。我们分析北大肿瘤医院单医生组肺癌前瞻性数据库2000年1月-2013年6月1, 290例NSCLC手术中病理分期为N2-Ⅲa期的121例患者，着重讨论淋巴结转移状态与远期预后的关系，探讨影响N2-Ⅲa期患者远期生存的因素。

## 1 材料和方法

### 1.1 患者资料

2000年1月-2013年6月本数据库共有手术NSCLC 1, 290例，其中根治性手术944例。入组标准：①有病理的原发性NSCLC；②按国际抗癌联盟（Union for International Cancer Control, UICC）/美国癌症联合会（American Joint Committee on Cancer, AJCC）2009年第7版TNM（tumor-node-metastasis, TNM）标准分期为N2-Ⅲa期患者；③术前排除脑、肝、骨、肾上腺转移；④解剖性肺叶或叶以上切除；⑤R0切除；⑥行系统性淋巴结清扫，至少包括3站N2淋巴结（其中必须包括隆突下淋巴结）及3站N1淋巴结。排除标准：①围手术期死亡；②严重合并症；③既往恶性肿瘤病史者。符合条件者共121例。

### 1.2 临床资料

全组121例，男性82例（67.8%），女性39例（32.2%）；年龄37岁-78岁，中位58岁；吸烟者60例（49.6%），不吸烟者61例（50.4%）。位于右肺上叶者42例（34.7%），右肺中叶者8例（6.6%），右肺下叶者28例（23.1%），左肺上叶者29例（24.0%），左肺下叶者14例（11.6%）。按2004年第4版世界卫生组织（World Health Organization, WHO）肺癌组织学分类以及2012年肺腺癌新分类标准，鳞癌24例（19.8%），腺癌88例（72.7%），其他9例（7.4%，包括腺鳞癌4例，大细胞癌5例）；病理可见脉管癌栓者38例（31.4%），未见脉管癌栓者83例（68.6%）。按UICC/AJCC 2009年第7版TNM标准分期，T1者34例（28.1%），T2者76例（62.8%），T3者11例（9.1%）（表 1）。

**1:** 121例N2-Ⅲa期NSCLC患者临床资料 Clinical characteristics of 121 patients with pathological N2-Ⅲa NSCLC

Variables	Value	Percentage
Age (yr)		
Median age	58	-
Age range	37-78	-
Gender		
Male	82	67.8%
Female	39	32.2%
Smooking index		
≤400	77	63.6%
> 400	44	36.4%
Chemotherapy	100	82.6%
Neoadjuvant chemotherapy	40	33.1%
Adjuvant chemotherapy	93	76.9%
Tumor location		
RUL	42	34.7%
RML	8	6.6%
RLL	28	23.1%
LUL	29	24.0%
LLL	14	11.6%
Surgical approach		
MIL	77	63.6%
Conventional thoracotomy	44	36.4%
Operation mode		
Lobectomy/Bilobectoy/Sleeve lobectomy	117	96.7%
Pneumonectomy	4	3.3%
pT stage		
Ⅰ	34	28.1%
Ⅱ	76	62.8%
Ⅲ	11	9.1%
Histological types		
ADC	88	72.7%
SQCC	24	19.8%
Others	9	7.4%
Intravascularcancer emboli grade (SQCC)		
Yes	38	31.4%
No	83	68.6%
ADC: adenocarcinoma; SQCC: squamous cell carcinoma; RUL: right upper lobe; RML: right middle lobe; RLL: right lower lobe; LUL: left upper lobe; LLL: left lower lobe; MIL: minimally invasive lobectomy; NSCLC: non-small cell lung cancer.		

### 1.3 术前分期

包括胸部增强CT、颈部及锁骨上区B超、腹部B超、气管镜、头颅增强MRI及骨扫描。2008年以后部分患者除以上检查外还行PET/CT进行分期（41例），其中纵隔镜分期者10例。

### 1.4 N2-Ⅲa期分类

根据N2转移站数分为单站N2与多站N2。根据2003年美国胸科医师学院的分类标准^[2]^，将N2-Ⅲa期分为4类，Ⅲa1期指最后的病理检查偶然发现的N2；Ⅲa2期指术中发现的单站N2；Ⅲa3期指术前分期（纵隔镜、其它的淋巴结活检或PET/CT）发现的单站或多站N2；Ⅲa4期指巨块或固定的多站N2（CT上短径 > 2 cm）。

### 1.5 手术与淋巴结清扫

均为解剖性R0切除，2008年前常规剖胸44例（36.4%）；之后为微创入路共77例（63.6%）。行肺叶切除者87例（71.9%），复合肺叶切除者22例（18.2%），支气管/和肺血管袖状切除及成型者8例（6.6%），全肺切除者4例（3.3%）。右肺常规清扫2R、4R、7、8R、9R、10R-13R组淋巴结；左肺常规清扫2L、4L、5、6、7、8L、9L、10L-13L组淋巴结，第12组与第13组淋巴结随所切肺叶共同送检。

### 1.6 围手术期化疗

术前新辅助化疗40例（33.1%），其中37例为术前病理明确或PET/CT诊断为N2；术后化疗93例（76.9%）。围手术期化疗方案为第三代化疗药加含铂两药方案，以TP方案为主（紫杉醇175 mg/m^2^ d1+顺铂75 mg/m^2^ d1-d3，21 d为1个周期）。

### 1.7 随访

门诊复查为主，术后2年内每3个月1次，3年-5年每半年1次，5年以后每年1次。复查包括胸部增强CT、头颅增强核磁共振（magnetic resonance imaging, MRI）/CT、全身骨扫描、锁骨上区及腹部超声。必要时行支气管镜及全身PET/CT。对未及时来门诊就诊者给予电话随访，门诊及电话随访记录患者生存状态，有无复发转移及复发转移部位，并录入肺癌数据库。本组随访以手术时间为起始时间，截止日期为2014年5月1日或死亡，随访率96.9%；中位随访时间29.4个月（6.2-111.7）。

### 1.8 统计学方法

应用SPSS 19.0（Chicago, IL, USA）统计软件，采用*Kaplan-Meier*法计算生存并绘制生存曲线，选用*Log-rank*检验。将单因素有统计学意义或存在差异趋势的因素进行多因素*Cox*回归分析，以*P* < 0.05为差异有统计学意义。

## 2 结果

### 2.1 N2分类

全组病理单站N2者79例（65.3%），多站N2者42例（34.7%，2站N2者30例，3站N2者8例，4站N2者4例）；Ⅲa1/a2期者42例（34.7%），Ⅲa3/a4期者79例（65.3%）。

### 2.2 单因素分析结果

全组5年生存率为43.6%，中位生存时间50.3个月。分层显示单站N2与多站N2的5年生存率分别为58.3%和25.5%（*P*=0.001，图 1）；Ⅲa1/a2期者与Ⅲa3/a4期者5年生存率分别为52.7%和38.4%（*P*=0.020，图 2）。腺癌（*n*=88）中位生存时间为59.8个月（45.8-73.8），非腺癌（*n*=33）中位生存时间为26.7个月（7.2-46.3），似乎腺癌要好于非腺癌者，但未达到统计学差异（*P*=0.057）；无脉管癌栓者（*n*=83）中位生存时间为61.2个月（46.3-76.1），有脉管癌栓者（*n*=38）中位生存时间为31.9个月（12.5-51.2），未达到统计学差异（*P*=0.089）。性别、年龄分层、吸烟、围手术期化疗、pT分期、肿瘤大小等因素并非本组预后因素（表 2）。

**1:**
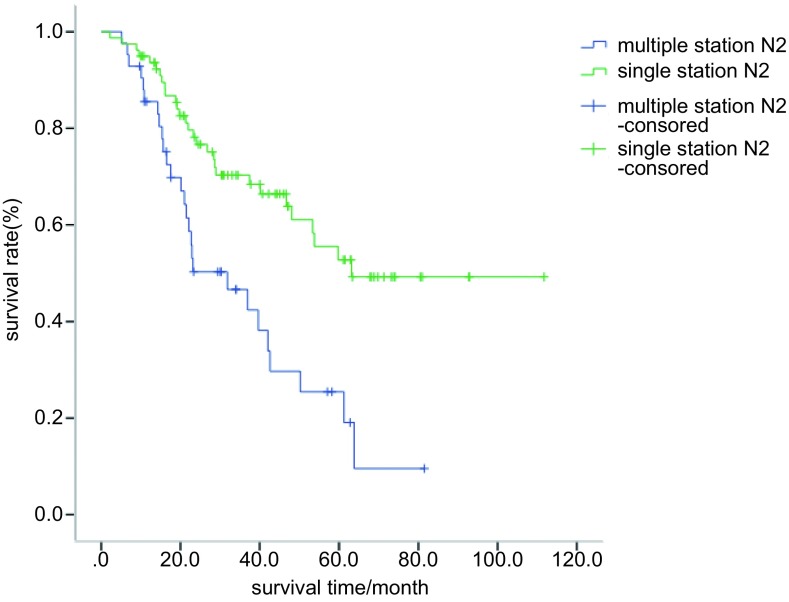
单站N2与多站N2 NSCLC患者（*n*=121）的*Kaplan-Meier*生存曲线，*P*=0.001。 Survival curves of patients with single station station N2 and multiple station N2. There was significant difference in 5-yr cumulative survival between the two groups (*P*=0.001).

**2:**
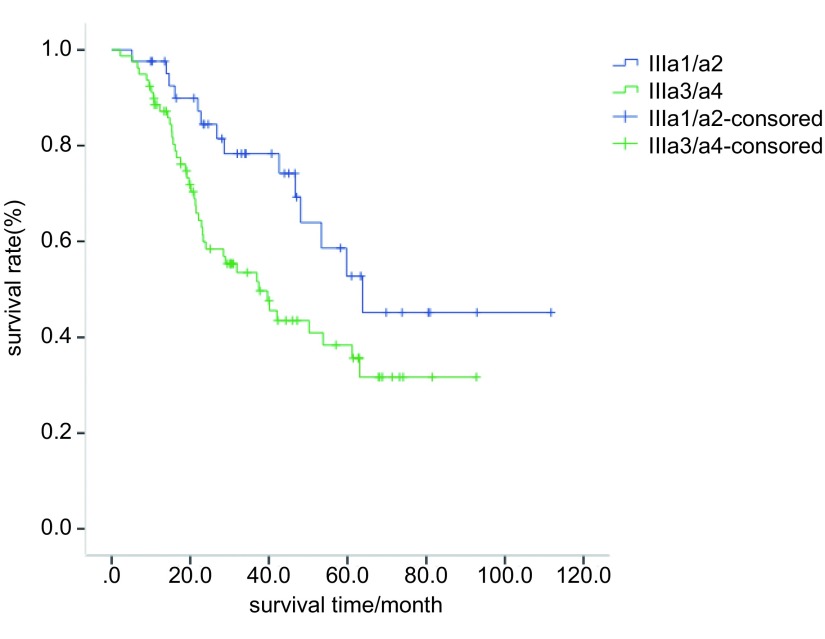
Ⅲa1/a2期与Ⅲa3/a4期NSCLC患者（*n*=121）的*Kaplan-Meier*生存曲线，*P*=0.020。 Survival curves of patients with Ⅲa1/a2 and Ⅲa3/a4. There was significant difference in 5-yr cumulative survival between the two groups (*P*=0.020).

**2:** 121例N2-Ⅲa期NSCLC患者单因素生存分析 Univariate analysis of N2-Ⅲa NSCLC patients for identifying prognostic factors

Items	*n*	Median survival time (mo)	Range	5-yr OS (%)	*P*
Gender					0.402
Male	82	42.6	31.4-53.8	37.7	
Female	39	63.1	57.1-69.1	55.4	
Age (yr)					0.836
< 65	88	53.4	34.8-72.0	43.2	
≥65	33	42.6	26.9-58.3	43.1	
Smoking index					0.363
< 400	77	59.8	42.3-77.2	52.8	
≥400	44	42.6	28.1-57.1	33.3	
Perioperative chemotherapy					0.258
Yes	100	48.1	34.4-61.8	40.0	
No	21	NA	NA	67.3	
Incision					0.118
Minimally invasive	77	59.8	37.1-82.5	49.1	
Thoracotomy	44	28.9	4.87-53.0	34.6	
Histological type					0.057
Adenocarcinoma	88	59.8	45.8-73.8	47.4	
Non-adenocarcinoma	33	26.7	7.2-46.3	34.5	
Intravascular cancer					0.089
Yes	38	31.9	12.5-51.2	23.1	
No	83	61.2	46.3-76.1	52.3	
Tumor diameter (cm)					0.269
≤3	50	50.3	28.2-72.4	44.1	
> 3	71	42.6	15.0-70.2	42.5	
N2 level					0.001
Single N2	79	63.1	NA	58.3	
Multiple N2	42	31.9	14.5-49.2	25.5	
N2 classification					0.020
Ⅲa1/a2	42	63.8	NA	52.7	
Ⅲa3/a4	79	37.5	24.0-50.9	38.4	
OS: overal survival; NA: not applicable.					

### 2.3 多因素分析结果

本组研究单因素分析显示仅N2淋巴结转移状态是影响预后的因素，但考虑到吸烟、围手术期化疗、脉管癌栓，肿瘤大小为临床普遍认可的影响预后因素，故也一并纳入到多因素*Cox*回归分析中。结果显示：以上因素中，仅单站N2转移（HR=0.326, 95%CI: 0.186-0.572, *P* < 0.001）与Ⅲa1/a2期（HR=0.494, 95%CI: 0.259-0.941, *P*=0.032）是影响N2-Ⅲa期患者术后远期生存的独立因素（表 3）。

**3:** N2-Ⅲa期NSCLC多因素*Cox*回归分析 Multivariate analysis of N2-Ⅲa NSCLC patients for identifying prognostic factors

Item	*P*	HR	95%CI
Age (< 65 yr *vs* ≥65 yr)	0.568	0.839	0.459-1.533
Gender (male *vs* female)	0.724	1.131	0.571-2.243
Smoking index (≥400 *vs* < 400)	0.641	1.162	0.619-2.181
Perioperative chemo (no *vs* yes)	0.207	1.733	0.738-4.069
Intravascularcancer (yes *vs* no)	0.104	1.637	0.904-2.966
Tumor diameter (> 3 cm *vs* ≤3 cm)	0.624	1.162	0.638-2.115
Histology (adeno *vs* non-adeno)	0.128	0.583	0.291-1.168
N2 level (single *vs* multiple)	< 0.001	0.326	0.186-0.572
pN2 classification (Ⅲa1/a2 *vs* Ⅲa3/a4)	0.032	0.494	0.259-0.941

## 3 讨论

N2-Ⅲa期NSCLC约占NSCLC的20%-30%，分期属于T1-3N2M0。目前认为该期存在异质性，既有术后镜下才发现的隐匿性N2，也有影像学上已肿大的单站N2或多站N2，还包含了多站纵隔淋巴结融合成团的N2。如何筛选优势人群以采取包括手术、化疗、放疗在内的综合治疗是目前研究的热点和难点。比较普遍的观点认为，多站N2及融合成团的巨块型N2病变（短径 > 2 cm）预后差，5年生存率仅5%-25%，应以同步放化疗为宜。而隐匿性或单站N2往往存在手术完整切除可能，其预后更接近N1，在围手术期化疗的协同作用下，5年生存率可达45%^[3]^。完整切除肿瘤（R0）是获得长期生存的关键，评估完整切除者的可行性，尤其是肺叶切除即可完全切除肿瘤者，预后较好；而无法完整切除者（R1和R2），疗效不佳。Decaluwé等^[4]^N2-Ⅲa期NSCLC者R0切除后中位生存时间和5年生存率分别为49个月和43%，而R1和R2切除则分别为17个月和19.9%。Albain等^[5]^R0肺叶切除者预后明显优于放疗者，中位生存时间分别为33.6个月和21.7个月（*P*=0.002），5年生存率分别为36%和18%。Koshy等^[6]^汇总N2-Ⅲa期NSCLC不同治疗的结果，诱导放化疗+肺叶切除（R0）的5年生存率为33.5%，肺叶切除+术后辅助治疗为20.3%，同期放化疗为10.9%，以诱导放化疗+肺叶切除（R0）组效果最好。本组均为R0切除，5年生存率为43.6%，中位生存时间50.3个月，由此可见，N2-Ⅲa期者经严格术前分期，挑选可完整切除者，并给与适当全身治疗，能获得令人满意的远期疗效。

术中或术后病理发现N2和单站N2手术预后较好N2的不同情况决定不同的预后，故应对N2行进一步分组。Sloan-Kettering肿瘤中心Martini等^[7]^较早提出这一差异，单纯手术的400例N2者，术前即高度怀疑为N2者5年生存率为9%；而术前无N2期病变证据者5年生存率为34%。Andre等^[8]^再次确认临床N2术后5年生存率为7%，镜下N2的5年生存率为29%，其中单站的镜下N2达34%。

Casali等^[9]^183例N2-Ⅲa期NSCLC术后全组5年生存率为20%，偶然N2者5年生存率为35.4%，临床N2者5年生存率为17.4%，单站N2者5年生存率为23.8%，多站N2者为14.7%。Riquet等^[10]^586例N2-Ⅲa期NSCLC中单站N2的5年生存率为32.2%，多站N2的5年生存率为19%，多站非融合N2的5年生存率为34%，融合N2者为13%。本组单站N2者（*n*=79, 65.3%）的5年生存率为58.3%，多站N2者（*n*=42, 34.7%）为25.5%（*P*=0.001）。Ⅲa1/a2期者（*n*=42, 34.7%）5年生存率为52.7%，Ⅲa3/a4期者（*n*=79, 65.3%）为38.4%（*P*=0.020）。单站N2完整切除的预后要好于多站N2者，生存更接近于N1期患者的预后；Ⅲa1/a2期患者预后亦要优于Ⅲa3/a4期患者。

既往研究^[11]^表明N2-Ⅲa期NSCLC患者行单纯手术疗效不佳，5年生存率仅为15%-20%，而以手术为主的多学科综合治疗尤其是围手术期化疗能提高患者的远期生存。对于N2-Ⅲa期NSCLC新辅助化疗的争论由来已久，最早的Ⅲ期随机临床试验是上世纪90年代由Rosell报道^[12]^，比较了新辅助化疗联合手术与单纯手术的远期疗效，术前化疗中位生存期高于单一手术，分别为20个月与10个月（*P* < 0.05）。Burdett等^[13]^的荟萃分析（*n*=2, 385）表明，新辅助化疗可降低Ia期-Ⅲa期患者10%的远处复发率，提高5%的5年生存率。

2003年后的国际肺癌辅助化疗试验（International Adjuvant Lung Cancer Trial, IALT）、加拿大国立癌症研究所试验（Canada National Cancer Institute, JBR10）、诺维本辅助化疗的国际试验（Ajuvant Navelbine International Trialist Association, ANITA）、美国肿瘤和白血病B组9633临床试验（Cancer and Leukemia Group B9633, CALGB9633）、肺癌的顺铂辅助化疗评估（Lung Adjuvant Cisplatin Evaluation, LACE）荟萃分析等逐步确立了术后辅助化疗的地位^[14-18]^。本组患者有82.6%（100/121）进行了围手术期的化疗，其中新辅助化疗40例（33.1%），辅助化疗93例（76.9%）。行新辅助化疗者中位生存时间39.7个月，而未行新辅助化疗者中位生存时间53.4个月，两者无明显差异（*P*=0.329），看似新辅助化疗并未带来生存获益，而且中位生存时间更短，但进一步分析发现本组新辅助化疗者多为治疗前即明确为N2病变者，即Ⅲa3期/Ⅲa4期者占92.5%（37/40），因此这一结果需要慎重解释；相反，本组总生存较好的原因中，与绝大多数患者（82.6%）接受了围手术期含铂方案化疗不无关系。

综上，N2-Ⅲa期NSCLC是一组异质性非常明显的疾病，其中单站N2转移者预后好于多站N2转移者，Ⅲa1/a2期患者预后好于Ⅲa3/a4期患者。高选择性N2-Ⅲa期NSCLC患者采取以外科手术为主的多学科综合治疗可获得较满意的远期生存。
